# Giant Left Ventricular Papillary Fibroelastoma Presented as Transient Ischemic Attack and Complicated by Post-Pericardiotomy Syndrome

**DOI:** 10.7759/cureus.12634

**Published:** 2021-01-11

**Authors:** Alaa Awad, Daire-Sean Gibbons, Hafiz Hussein, Sarra Mohamed, Sara Mohammed

**Affiliations:** 1 Internal Medicine, Royal College of Surgeons Hospitals Group, Cavan General Hospital, Cavan, IRL; 2 Cardiology, Beaumont Hospital, Dublin, IRL

**Keywords:** cardiac tumors, lv thrombus, tia, cardiac imaging, post-pericardiotomy syndrome

## Abstract

Papillary fibroelastoma is a benign cardiac tumour that most commonly presents as an incidental finding on imaging but may present with an acute neurological event due to embolic phenomena. We report a 51-year-old female who presented with focal neurology of the right hand that lasted for 30 minutes. Her initial investigations including CT-brain were unremarkable, and given her low-risk profile for stroke she was discharged for routine outpatient workup of possible transient ischaemic attack. Transthoracic echo detected a large mobile mass attached to the left ventricular wall. This was mistakenly diagnosed as a left ventricular thrombus, for which she was commenced on warfarin. After three months on warfarin without reduction in the size of the mass, cardiac MRI was performed. The scan was repeated as the initial imaging failed to demonstrate the tumour. This was followed by positron emission tomography which suggested a benign mass of the left ventricle. The patient underwent surgical excision of the tumour and developed post-pericardiotomy syndrome. Histopathology confirmed papillary fibroelastoma. Though rare, cardiac neoplasm may remain a differential diagnosis for acute neurological presentations in non-classical patients.

## Introduction

Papillary fibroelastoma (PFE) is one of the benign, primary, cardiac neoplasms, and accounts for <10% of all these tumors. They are small neoplasm of valve apparatus usually found incidentally on imaging, such as echocardiography, or at autopsy [1]. Though they are benign, recent studies have demonstrated high incidence of embolic events that may lead to life-threatening complications [2]. Here we present a case of giant left ventricular (LV) PFE that manifested as transient ischemic attack (TIA) and complicated by post-pericardiotomy syndrome (PPS) after surgical resection.

## Case presentation

**Figure 1:**
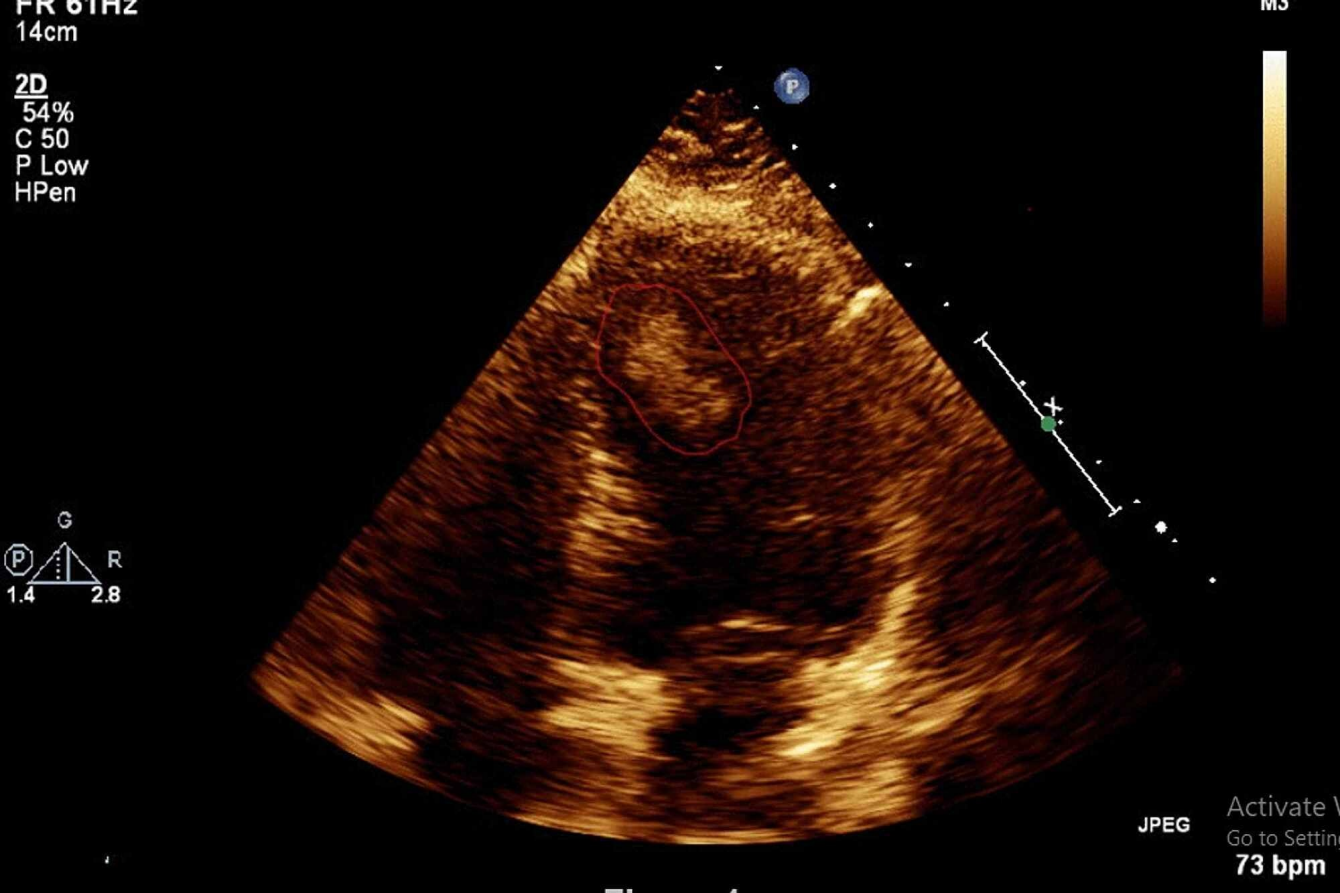
Transthoracic Echocardiography showing apical/septal mass in the LV (apical/four-chamber view)

**Figure 2:**
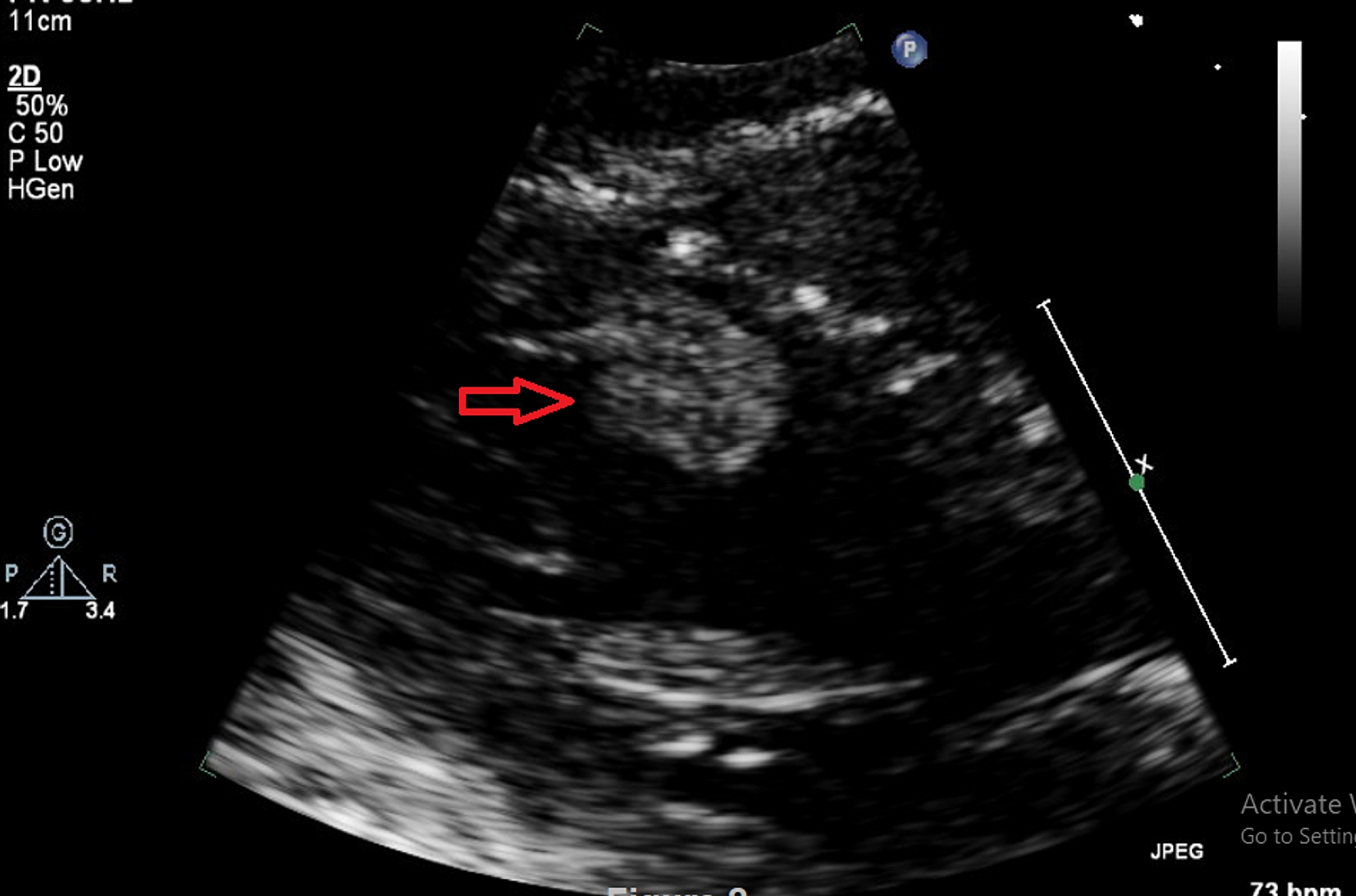
Transthoracic Echocardiography showing apical/septal mass in the LV (long axis, focused LV view)

A 51-year old lady presented to her local hospital emergency department with an episode of right-hand weakness which came on during office work and lasted for 30 minutes. There were no other lateralization signs. She denied any cardiac symptoms, previous embolic event, miscarriage, or any history suggestive of vasculitis. Her systematic review, past medical history, family history, drug and social history were non-contributory. The clinical examination revealed no neurological deficits and the other systems examination were unremarkable. Computerized tomography (CT) showed no acute pathology. Routine blood work including full blood count, renal function, electrolytes, coagulation, and thyroid function test were all normal, excepting a mildly elevated cholesterol and borderline D-dimer. A provisional diagnosis of TIA was considered, and she was commenced on aspirin and a statin. The following investigations were arranged as an outpatient workup for stroke/TIA: Holter Monitor, Carotid Doppler and Transthoracic Echocardiography (TTE). During outpatient work up the patient remained symptom free. Holter Monitor, Carotid Dopplers, repeat electrocardiogram (ECG) and chest X-ray (CXR) were all unremarkable. However, TTE revealed a dense mobile mass measuring 2.6 x 1.7 cm attached to the distal septum of the LV suspicious with thrombus (Figure 1, 2), as well as normal LV systolic function, normal wall motion, and intact cardiac valves.

**Figure 3:**
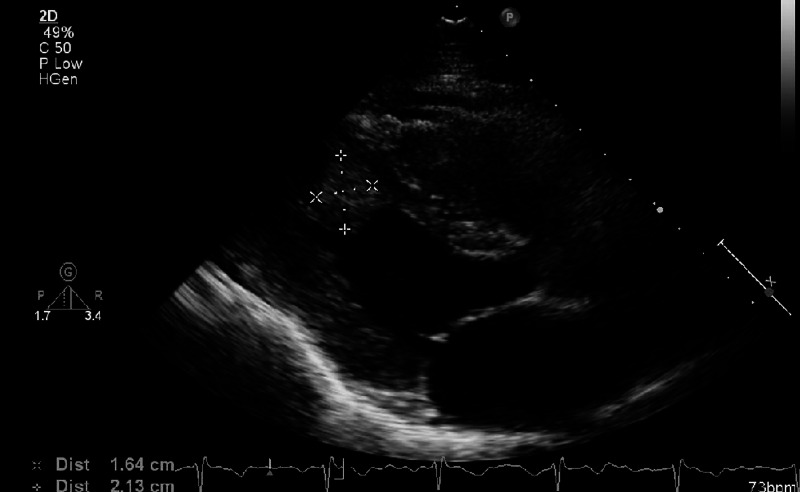
Dense mobile structure noted measuring 2.37x1.38cm at distal septum/apex suggestive of thrombus.

The patient was diagnosed with an LV mural thrombus, initiated on anticoagulation with warfarin, target international normalized ratio (INR) 2-3, and admitted for further inpatient workup. Without obvious wall motion abnormalities or arrhythmia, the patient was investigated for secondary causes of thrombus in the form of thrombophilia screen, tumour markers, and CT thorax abdomen and pelvis for evidence of malignancy. Again these investigations revealed no abnormalities. The patient was discharged and outpatient cardiac magnetic resonance imaging (MRI) was arranged. Repeat TTE after three months of anticoagulation showed no reduction in the size of the LV mass (Figure 3).

**Figure 4:**
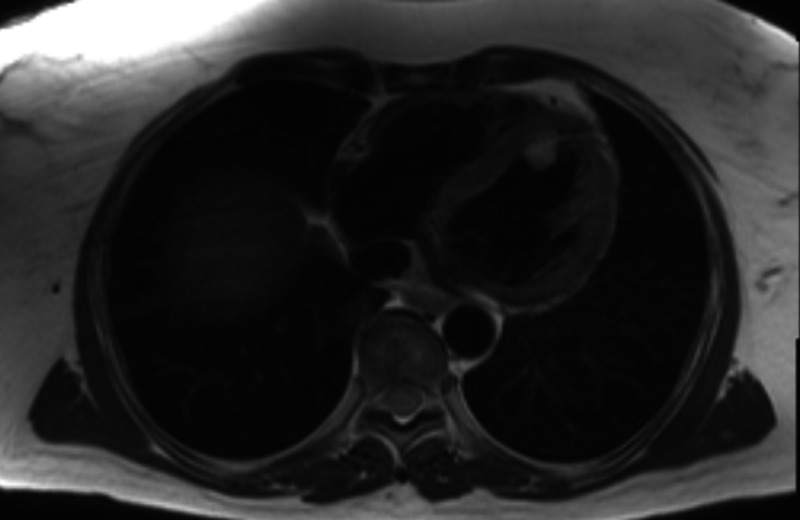
Cardiac MRI transverse view- clearly demonstrated LV apical septal mass

**Figure 5:**
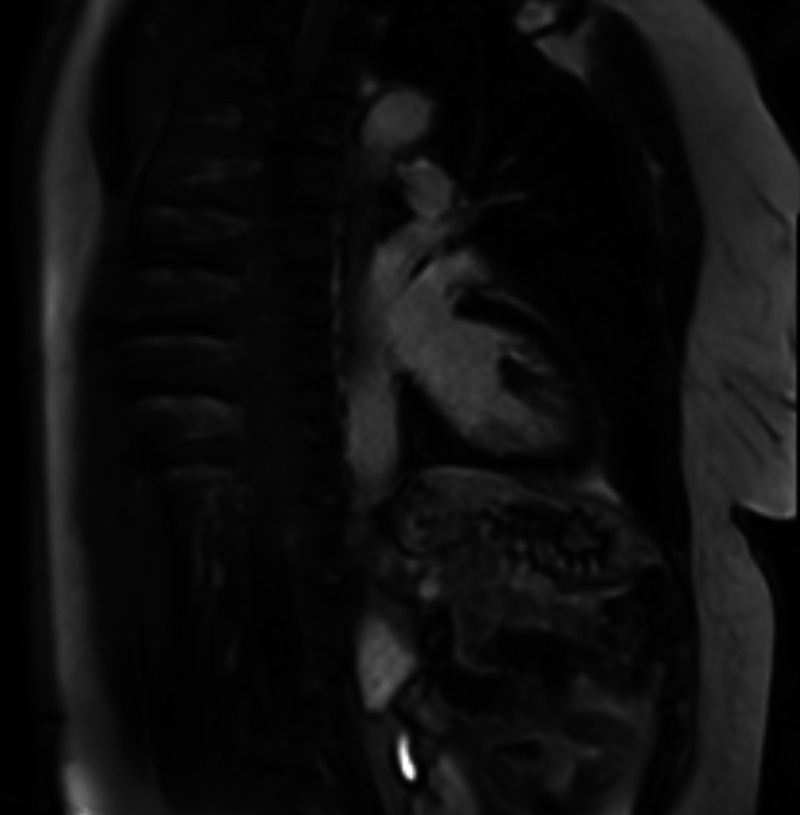
Cardiac MRI sagittal view- clearly demonstrated LV apical septal mass

Surprisingly, an initial cardiac MRI reported no abnormal findings based on misapplied protocol for presumed apical rather than a septal mass. Given the contradictory result, this was repeated with particular focus on the septal wall on subsequent scanning and a 23 x 15.5mm mass adherent principally to anteroseptal wall at mid-ventricular level was identified (Figure 4, 5).

**Figure 6:**
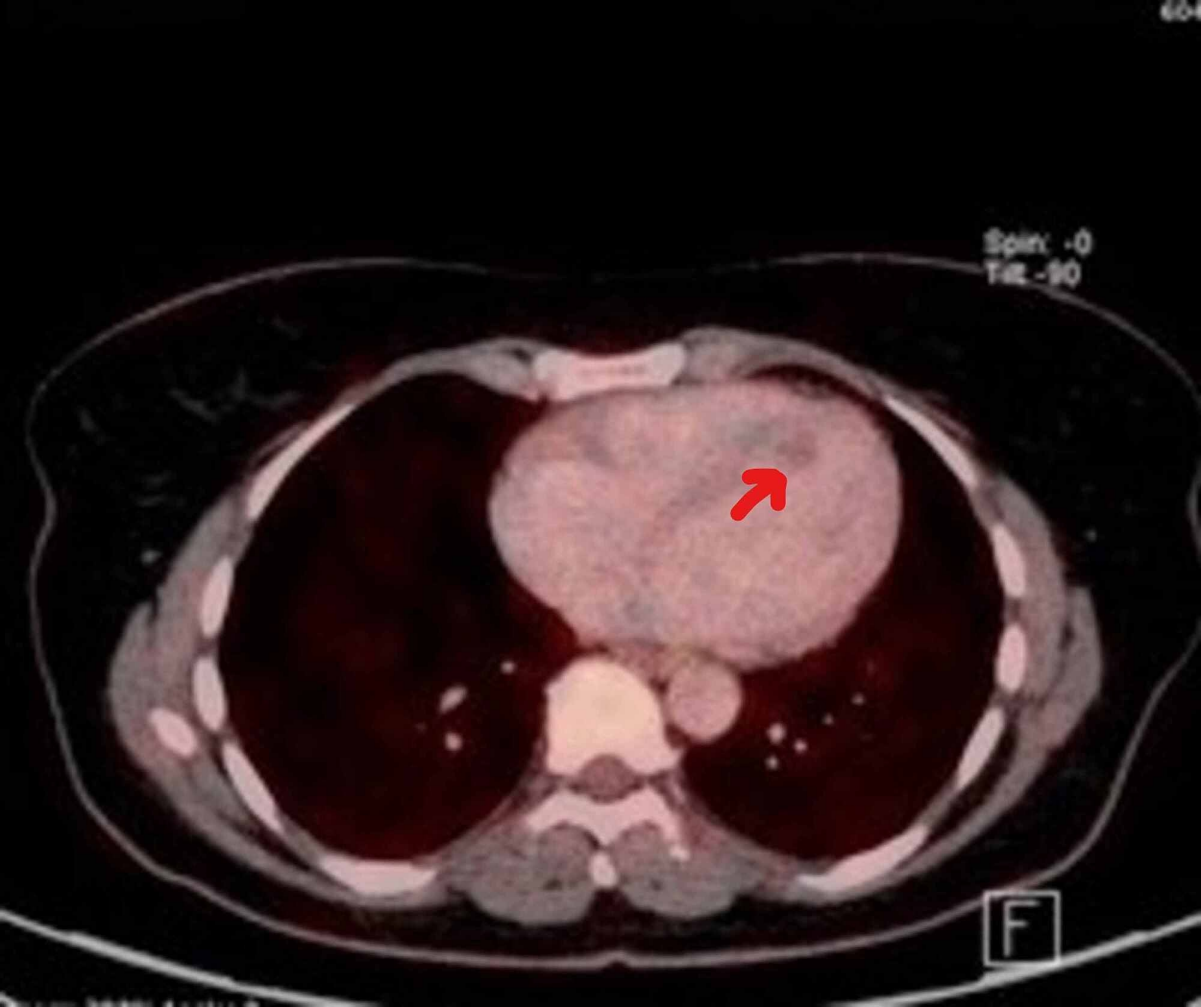
Positron emission tomography (PET) imaging showing low uptake and low metabolic activity of LV mass

**Figure 7:**
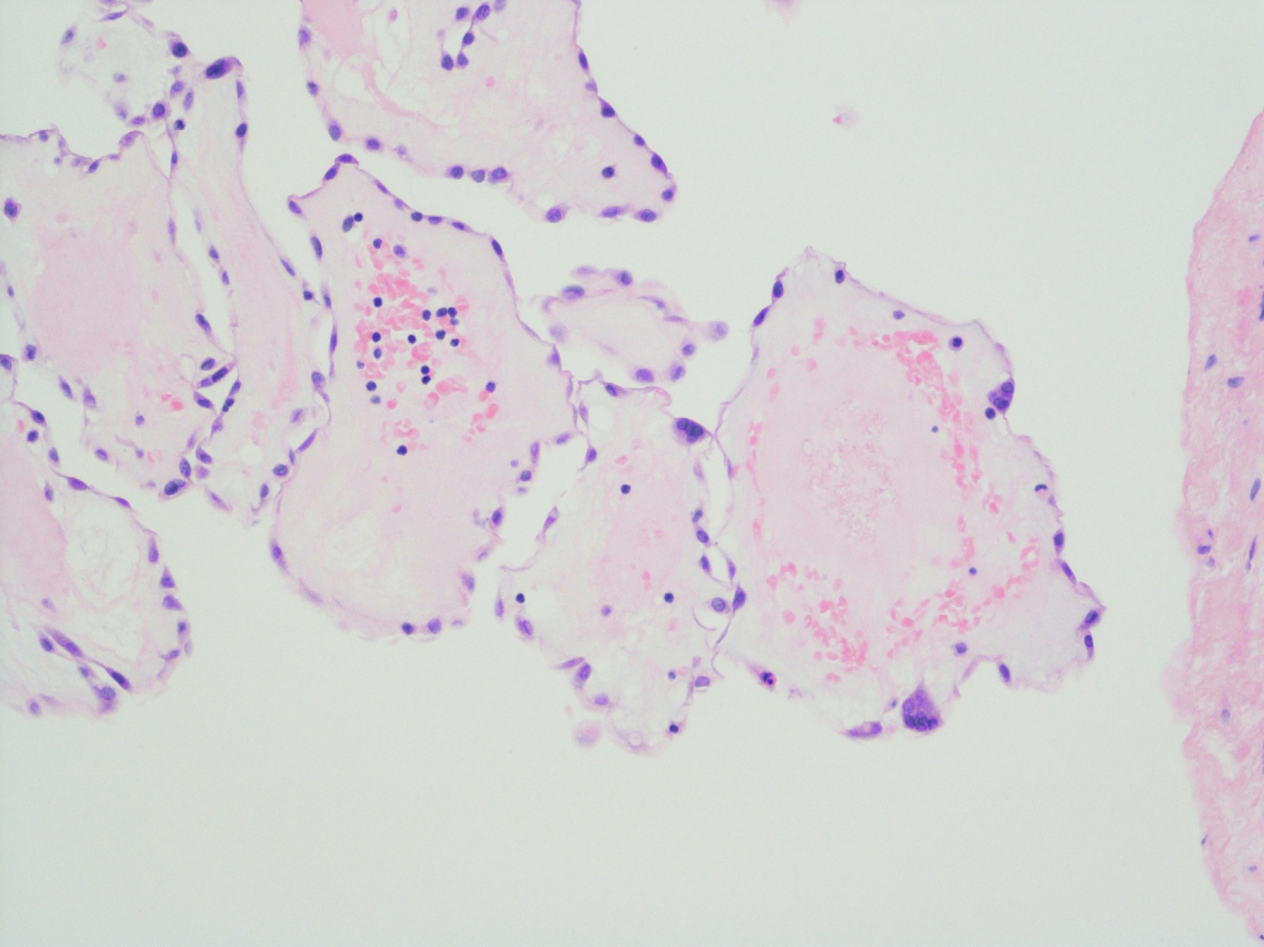
Histopathology consistent with Papillary Fibroelastoma

**Figure 8:**
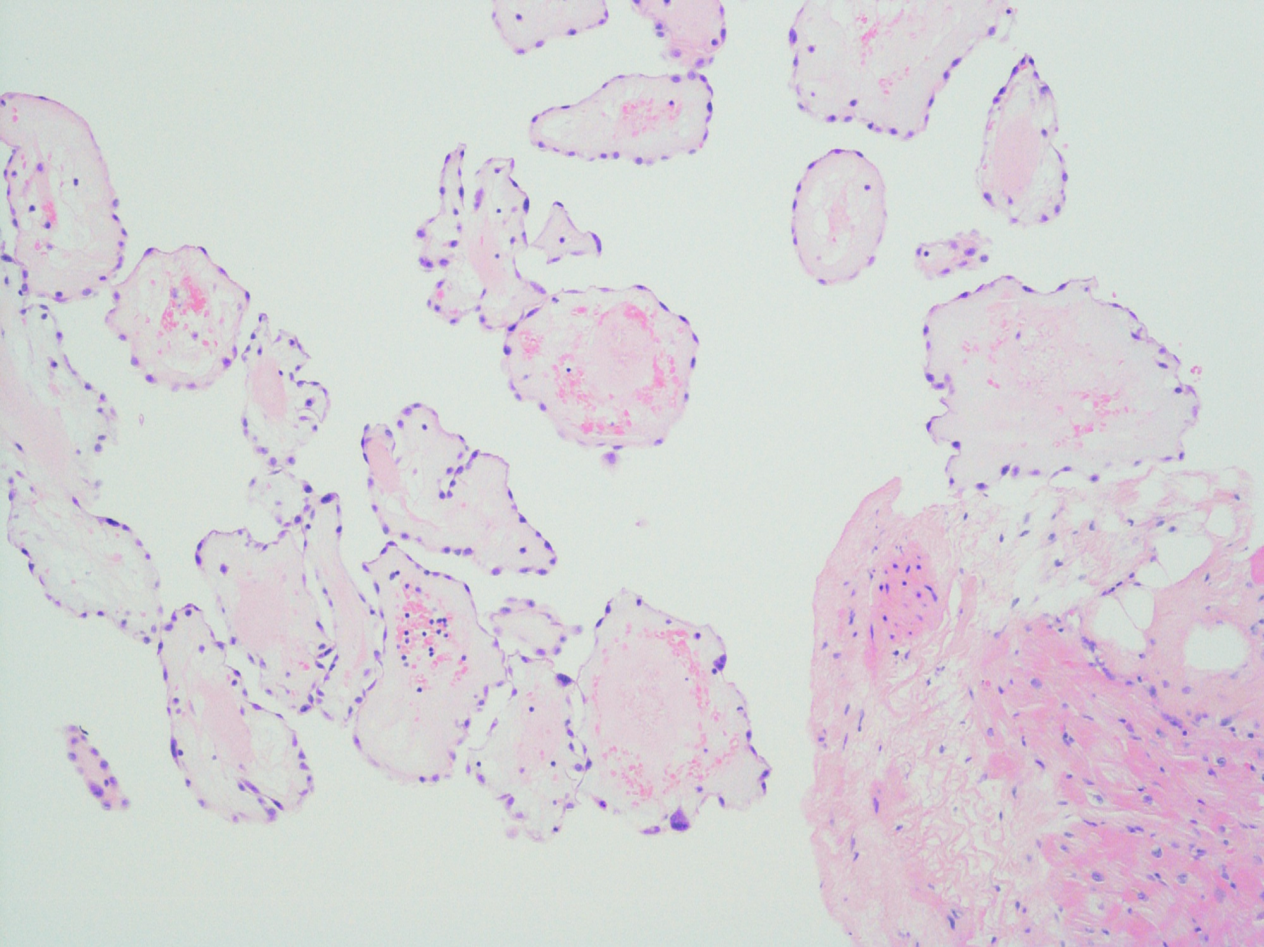
Histopathology consistent with Papillary Fibroelastoma

This was followed by positron emission tomography (PET) which confirmed the lesion location and suggested low metabolic activity favoring a benign lesion (Figure 6). The patient was referred to cardio-thoracic surgery and an urgent elective resection of the tumour was successfully performed. The mass was sent for histopathology and subsequent histopathology confirmed a diagnosis of cardiac papillary fibroelastoma (Figure 7, 8).

**Figure 9:**
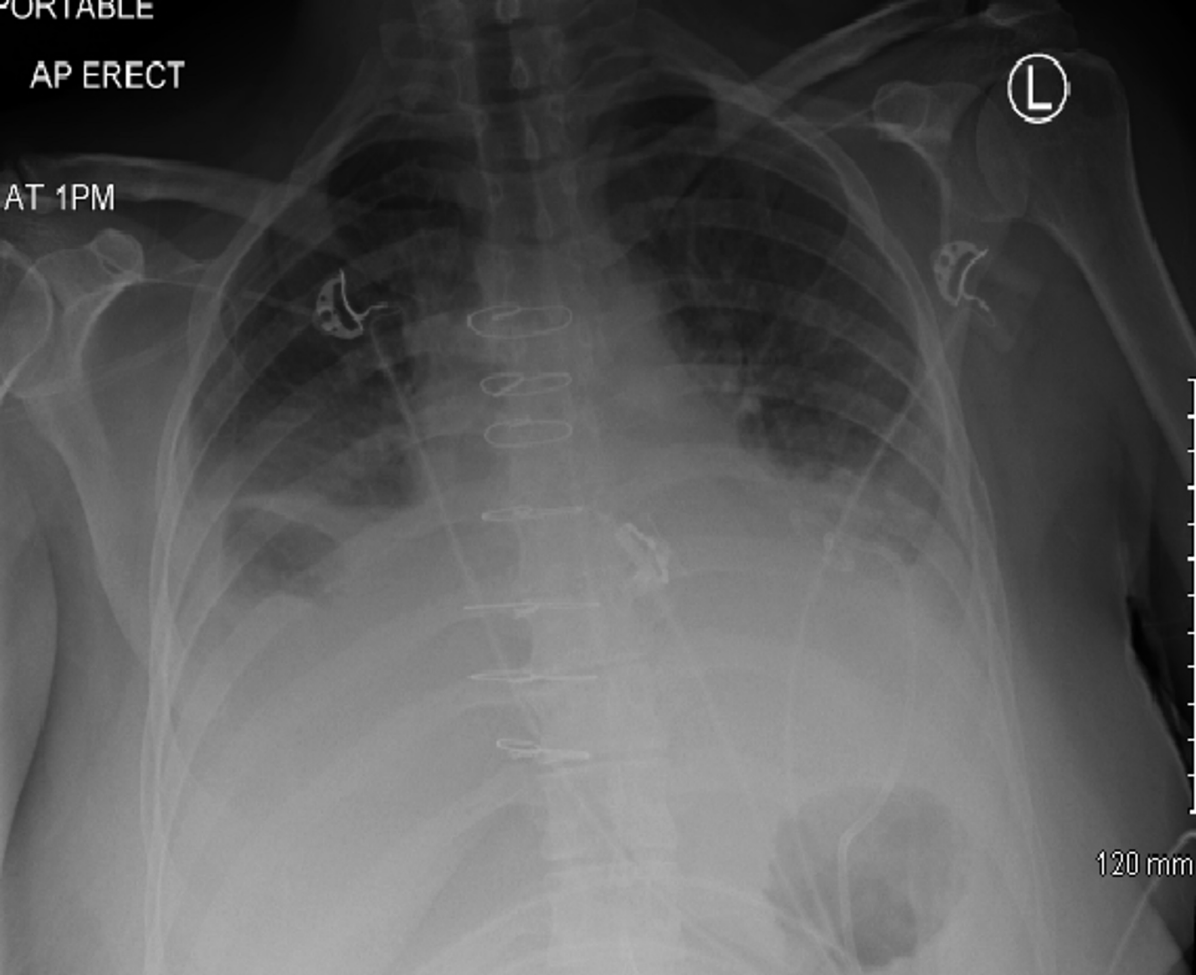
Chest X-ray (CXR) showing cardiomegaly and bilateral plural effusion

Five weeks after operation, the patient represented to the Emergency Department clinically unwell complaining of episodes of shortness of breath with increasing frequency and severity accompanied by chest discomfort. On assessment, she was hemodynamically unstable with low blood pressure (54/39), tachycardia, and low oxygen saturation. Cardiovascular examination revealed raised jugular venous pressure (JVP), muffled heart sounds and bilateral pedal edema. Investigations showed severe cardiomegaly on CXR (Figure 9), low voltage QRS complexes on ECG, acute kidney injury, and deranged liver function with a supra-therapeutic INR of 10. An urgent TTE detected massive life-threatening cardiac tamponade.

**Figure 10:**
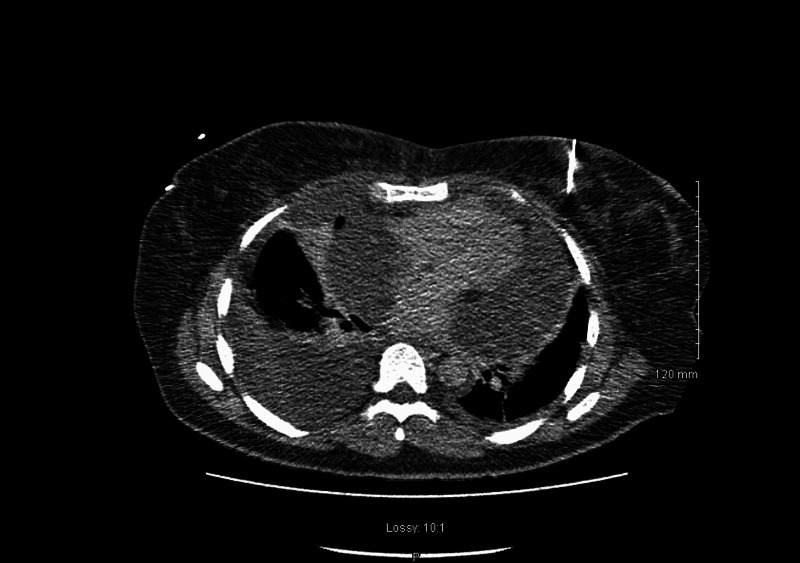
CT thorax showing large volume pericardial effusion with tamponade and bilateral plural effusion more prominent on right lung.

Immediate CT-guided pericardiocentesis drained 900 ml of serous fluid (Figure 10). She was then admitted directly to the intensive care unit and later transfer to a cardiothoracic center where she was monitored and received further non-surgical management. One week later, with a diagnosis of post-pericardiotomy syndrome, the patient was discharged home, clinically well and on a course of oral steroids and colchicine.

## Discussion

Primary cardiac tumours are considered to be rare, with a prevalence of 0.001-0.3% according to autopsy series done in UK [3]. Further, PFEs probably represent less than 10% of these cardiac tumours. However, like many rare pathologies, the diagnosis of cardiac tumours is increasing in line with the use of advanced imaging modalities. Though myxomas were long considered to be the most prevalent primary cardiac tumour across all age groups, a recent case series by Tamin et al. at the Mayo Clinic now suggests that PFEs are the most common benign cardiac neoplasm of adulthood [4]. PFE is primarily a valvular tumour. It most commonly arises from the aortic valve, but may also be found on the mitral valve [4-5]. Multifocal and non-valvular sites are considered to be extremely rare with a preponderance for the left ventricular cavity [5].

The presence of this tumour in the LV can be challenging to differentiate from other intracardiac pathologies. Though our patient had no underlying ischemic heart disease, she was managed as LV thrombus. The fact that she presented with an embolic event, together with the location and hypermobility of the mass, supported the presumed diagnosis.

Histologically, PFEs are avascular frondlike structures that are composed of fibroelastic tissue surrounded by endocardium [6]. The pathogenesis of PFEs remains unclear as to whether it is a neoplastic process, hamartomatous lesions, or reactive growth [7]. It has also been suggested that PFEs represent giant forms of Lambl’s excrescence, evolving from a thrombotic phenomenon secondary to traumatization of the endothelial cells. This hypothesis is supported by their propensity for embolization [8].

For symptomatic patients, the most common presentation of PFE reported in the literature is a cerebrovascular event [1-4]. This can arise from either a fibrin thrombus or from embolism of the tumour itself. Depending on their location, dimensions, and mobility, direct occlusion of cardiac structures may also occur [1-2]. This can give rise to a wide range of symptoms, from focal neurology to dyspnea, angina, or myocardial infarction [1]. Left cardiac tumours have a higher tendency for thromboembolism as reported in several surgical series [5]. However, some studies reported no significant association between pathological-anatomic characteristics of PFEs and embolism [4-7].

Cardiac MRI has well-known advantages as a diagnostic tool over echocardiography due to its exquisite sensitivity to tissue differentiation. In spite of this, cardiac MRI struggled in this case to identify a mass seen easily on TTE and ultimately required multiple imaging sessions and expert consultation and interpretation. In contrast, TTE picked up the tumour easily, providing valuable information about its location, size, and motion, but ultimately delayed the patient’s diagnosis due to a lack of sensitivity to the tissue structure of the mass. It is reasonable to conclude that echocardiography remains the modality of choice for the initial imaging investigation of heart structure and function but that in non-classical cases advanced imaging such as CMR and PET scanning may provide additional insight [9].

This patient had a stormy course and five weeks after cardiac surgery she developed post-pericardiotomy syndrome manifesting as a life-threatening cardiac tamponade with hypovolemic shock, multi-organ failure, and elevated INR. PPS is one of the fatal consequences and least discussed complications of cardiac surgery. It carries an incidence of 10-40% [10] and is hypothesized to be due to pericardial injury and subsequent inflammation. Colchicine has been shown to be effective in both treating and preventing the development of PPS [10] and was likely beneficial to the patient in this case.

## Conclusions

We report a middle-aged woman without obvious cardiovascular risk factors who presented with TIA related to underlying benign cardiac PFE tumor that was confirmed by histopathology after surgical resection. Echocardiography was of value as a first choice imaging modality. It successfully identified the source of her neurological deficit as a left ventricular mass giving rise to an embolism. However, advanced imaging including cardiac MRI and PET scanning was necessary to correctly identify her cardiac pathology as a benign tumour necessitating prompt surgical intervention. Serious postoperative complications of cardiothoracic surgery are common and both patient and clinicians should be aware of symptoms suggestive of post-pericardiotomy syndrome.

This case emphasizes that in non-classical patients without obvious risk factors for cardiovascular disease or with poor responses to standard therapies, an open mind and advanced imaging can be more than a fishing exercise.
